# Supplementary figures and images for: Bacteroides thetaiotaomicron Fosters the Growth of Butyrate-Producing Anaerostipes caccae in the Presence of Lactose and Total Human Milk Carbohydrates
Source: Microorganisms. 2020 Oct 1;8(10):1513. doi: 10.3390/microorganisms8101513 (PMC7601031; doi:10.3390/microorganisms8101513)

(A)

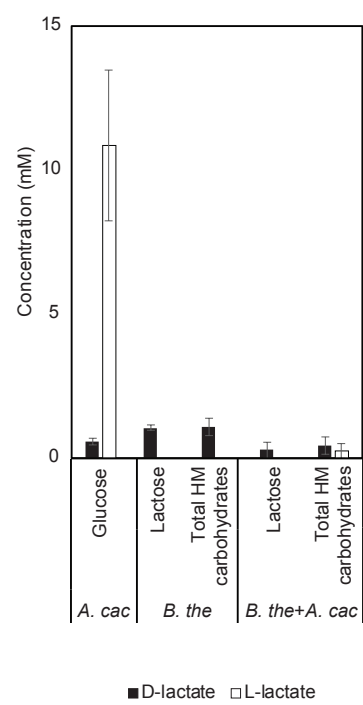

(B)

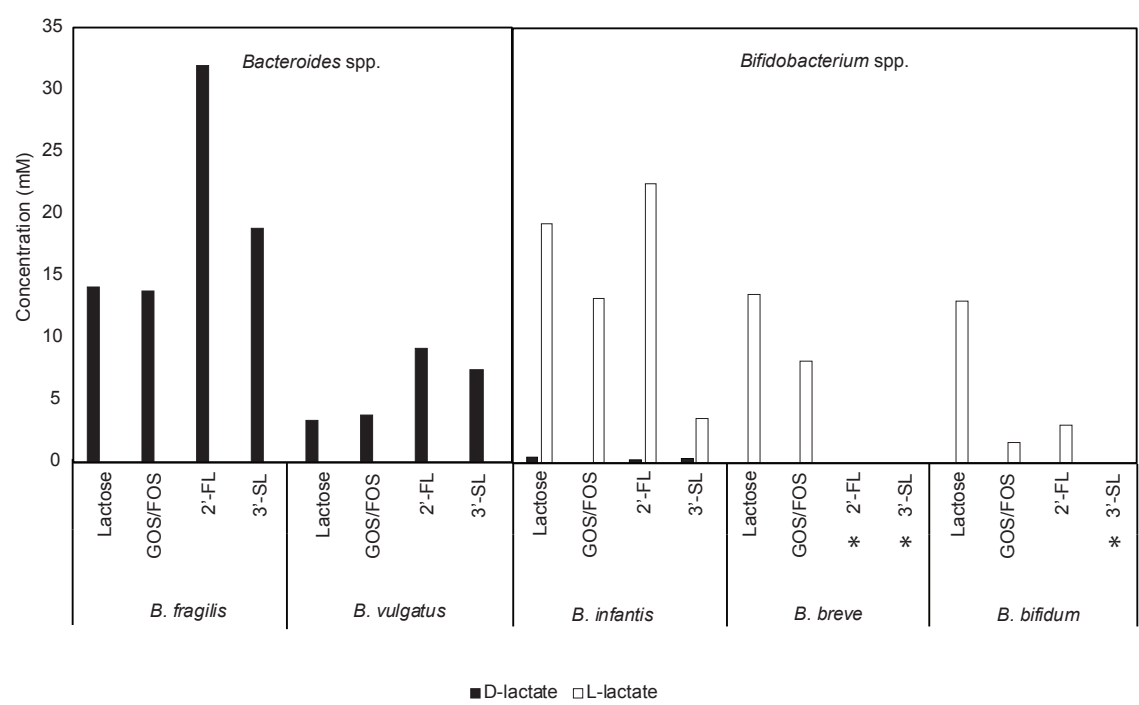

Supplement: Supplementary file 1 [file microorganisms-08-01513-s001.zip › microorganisms-919166-supplementary/Figure S2.pdf]
