# Supplementary material for: Bacteroides thetaiotaomicron Fosters the Growth of Butyrate-Producing Anaerostipes caccae in the Presence of Lactose and Total Human Milk Carbohydrates
Source: Microorganisms. 2020 Oct 1;8(10):1513. doi: 10.3390/microorganisms8101513 (PMC7601031; doi:10.3390/microorganisms8101513)
Supplement: Supplementary file 1 [file Figure S1.pdf › microorganisms-919166-supplementary/Figure S1.pdf]

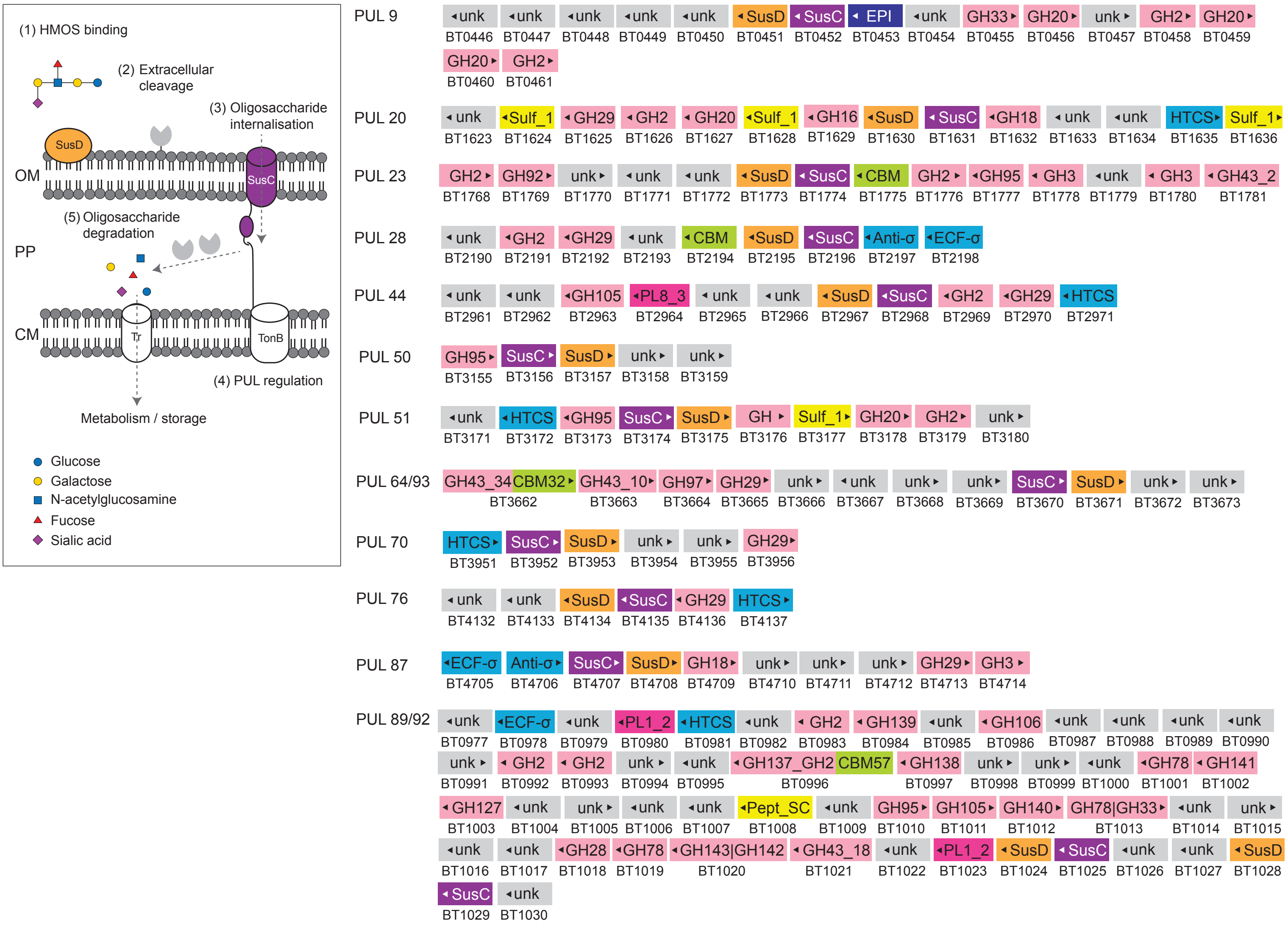

(1) HMOS binding
(2) Extracellular cleavage
(3) Oligosaccharide internalisation
SusD
SusC
OM
(5) Oligosaccharide degradation
PP
CM
Tr
TonB
(4) PUL regulation
Metabolism / storage
Glucose
Galactose
N-acetylglucosamine
Fucose
Sialic acid
PUL 9
unk unk unk unk unk SusD SusC EPI unk GH33 GH20 unk GH2 GH20
BT0446 BT0447 BT0448 BT0449 BT0450 BT0451 BT0452 BT0453 BT0454 BT0455 BT0456 BT0457 BT0458 BT0459
GH20 GH2
BT0460 BT0461
PUL 20
unk Sulf_1 GH29 GH2 GH20 Sulf_1 GH16 SusD SusC GH18 unk unk HTCS Sulf_1
BT1623 BT1624 BT1625 BT1626 BT1627 BT1628 BT1629 BT1630 BT1631 BT1632 BT1633 BT1634 BT1635 BT1636
PUL 23
GH2 GH92 unk unk unk SusD SusC CBM GH2 GH95 GH3 unk GH3 GH43_2
BT1768 BT1769 BT1770 BT1771 BT1772 BT1773 BT1774 BT1775 BT1776 BT1777 BT1778 BT1779 BT1780 BT1781
PUL 28
unk GH2 GH29 unk CBM SusD SusC Anti-σ ECF-σ
BT2190 BT2191 BT2192 BT2193 BT2194 BT2195 BT2196 BT2197 BT2198
PUL 44
unk unk GH105 PL8_3 unk unk SusD SusC GH2 GH29 HTCS
BT2961 BT2962 BT2963 BT2964 BT2965 BT2966 BT2967 BT2968 BT2969 BT2970 BT2971
PUL 50
GH95 SusC SusD unk unk
BT3155 BT3156 BT3157 BT3158 BT3159
PUL 51
unk HTCS GH95 SusC SusD GH Sulf_1 GH20 GH2 unk
BT3171 BT3172 BT3173 BT3174 BT3175 BT3176 BT3177 BT3178 BT3179 BT3180
PUL 64/93
GH43_34 CBM32 GH43_10 GH97 GH29 unk unk unk unk SusC SusD unk unk
BT3662 BT3663 BT3664 BT3665 BT3666 BT3667 BT3668 BT3669 BT3670 BT3671 BT3672 BT3673
PUL 70
HTCS SusC SusD unk unk GH29
BT3951 BT3952 BT3953 BT3954 BT3955 BT3956
PUL 76
unk unk SusD SusC GH29 HTCS
BT4132 BT4133 BT4134 BT4135 BT4136 BT4137
PUL 87
ECF-σ Anti-σ SusC SusD GH18 unk unk unk GH29 GH3
BT4705 BT4706 BT4707 BT4708 BT4709 BT4710 BT4711 BT4712 BT4713 BT4714
PUL 89/92
unk ECF-σ unk PL1_2 HTCS unk GH2 GH139 unk GH106 unk unk unk unk
BT0977 BT0978 BT0979 BT0980 BT0981 BT0982 BT0983 BT0984 BT0985 BT0986 BT0987 BT0988 BT0989 BT0990
unk GH2 GH2 unk unk GH137_GH2 CBM57 GH138 unk unk unk GH78 GH141
BT0991 BT0992 BT0993 BT0994 BT0995 BT0996 BT0997 BT0998 BT0999 BT1000 BT1001 BT1002
GH127 unk unk unk unk Pept_SC unk GH95 GH105 GH140 GH78|GH33 unk unk
BT1003 BT1004 BT1005 BT1006 BT1007 BT1008 BT1009 BT1010 BT1011 BT1012 BT1013 BT1014 BT1015
unk unk GH28 GH78 GH143|GH142 GH43_18 unk PL1_2 SusD SusC unk unk SusD
BT1016 BT1017 BT1018 BT1019 BT1020 BT1021 BT1022 BT1023 BT1024 BT1025 BT1026 BT1027 BT1028
SusC unk
BT1029 BT1030
